# Supplementary material for: Pervasive function and evidence for selection across standing genetic variation in S. cerevisiae
Source: Nat Commun. 2019 Mar 15;10:1222. doi: 10.1038/s41467-019-09166-1 (PMC6420628; doi:10.1038/s41467-019-09166-1)
Supplement: Supplementary file 1 — Supplementary Information [file 41467_2019_9166_MOESM1_ESM.pdf]

Supplementary Information

To accompany "Pervasive function and evidence for selection across standing genetic variation in *S. cerevisiae*" by Jakobson, She, and Jarosz.

Supplementary Figures

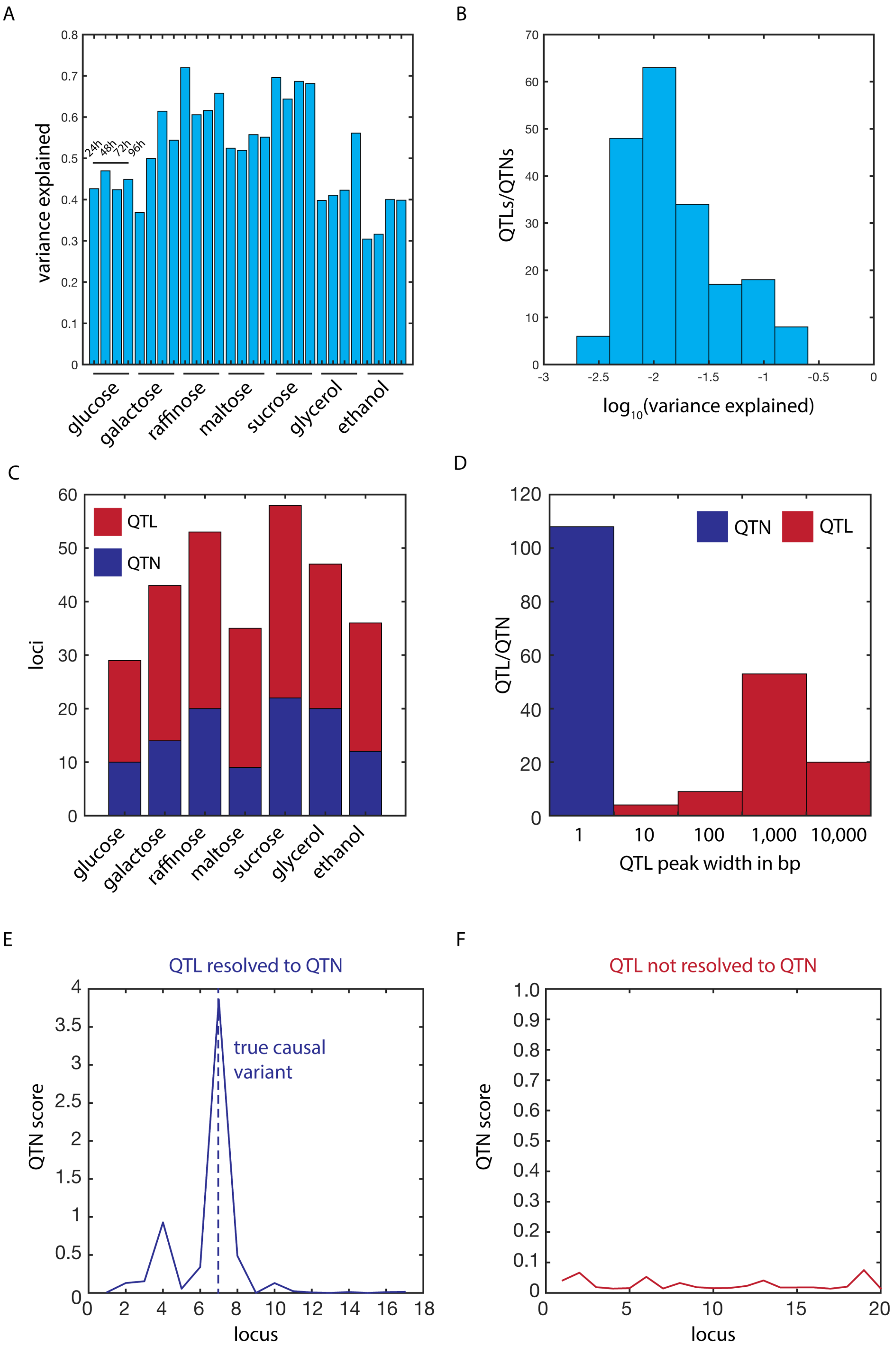

Supplementary Figure 1. (A) Total variance explained by our model for each trait examined. **(B)** Absolute variance explained for all statistically significant QTL across all traits examined. **(C)** Number of loci resolved (QTN) or not resolved (QTL) to single causal variant for each trait examined. **(D)** Histogram of the QTN score peak width in base-pairs for all QTL; peak width=1 represents the discovery of a single causal variant. Example plots of the QTN score metric for **(E)** a locus resolved to a single causal variant and **(F)** a locus not resolved to a single causal variant. Source data are provided as a Source Data file.

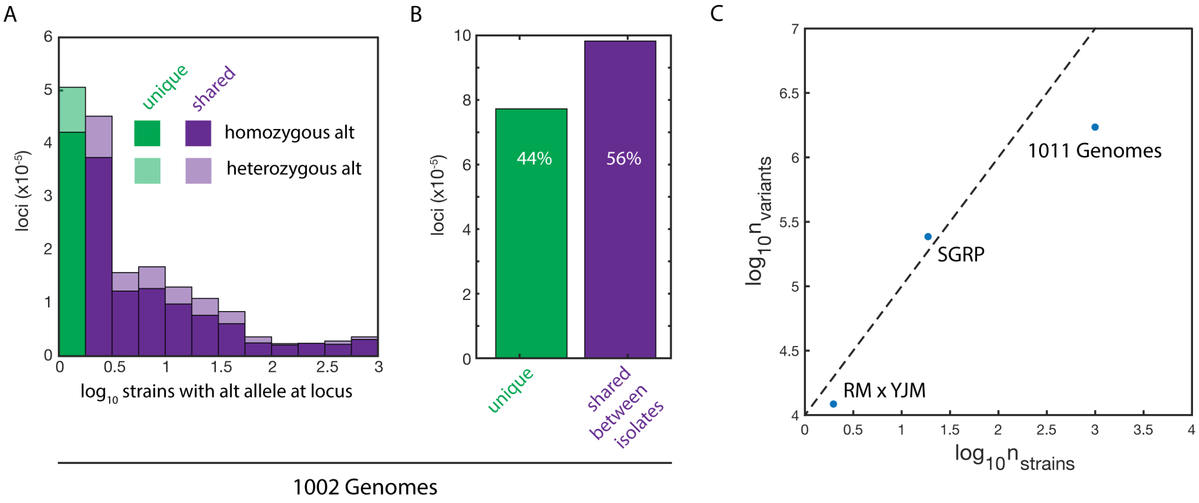

Supplementary Figure 2. (A) Histogram of the number of strains within the 1002 Yeast Genomes Project collection bearing an alternate allele at each locus for which a variant allele exists; loci with homozygous and heterozygous alternate alleles are shown separately; **(B)** Fraction of all variants segregating in the 1002 Yeast Genomes Project collection that are unique or shared with another 1002 Yeast Genomes Project isolate. **(C)** Number of segregating variants in each cross/strain collection plotted against number of strains in that cross/collection.

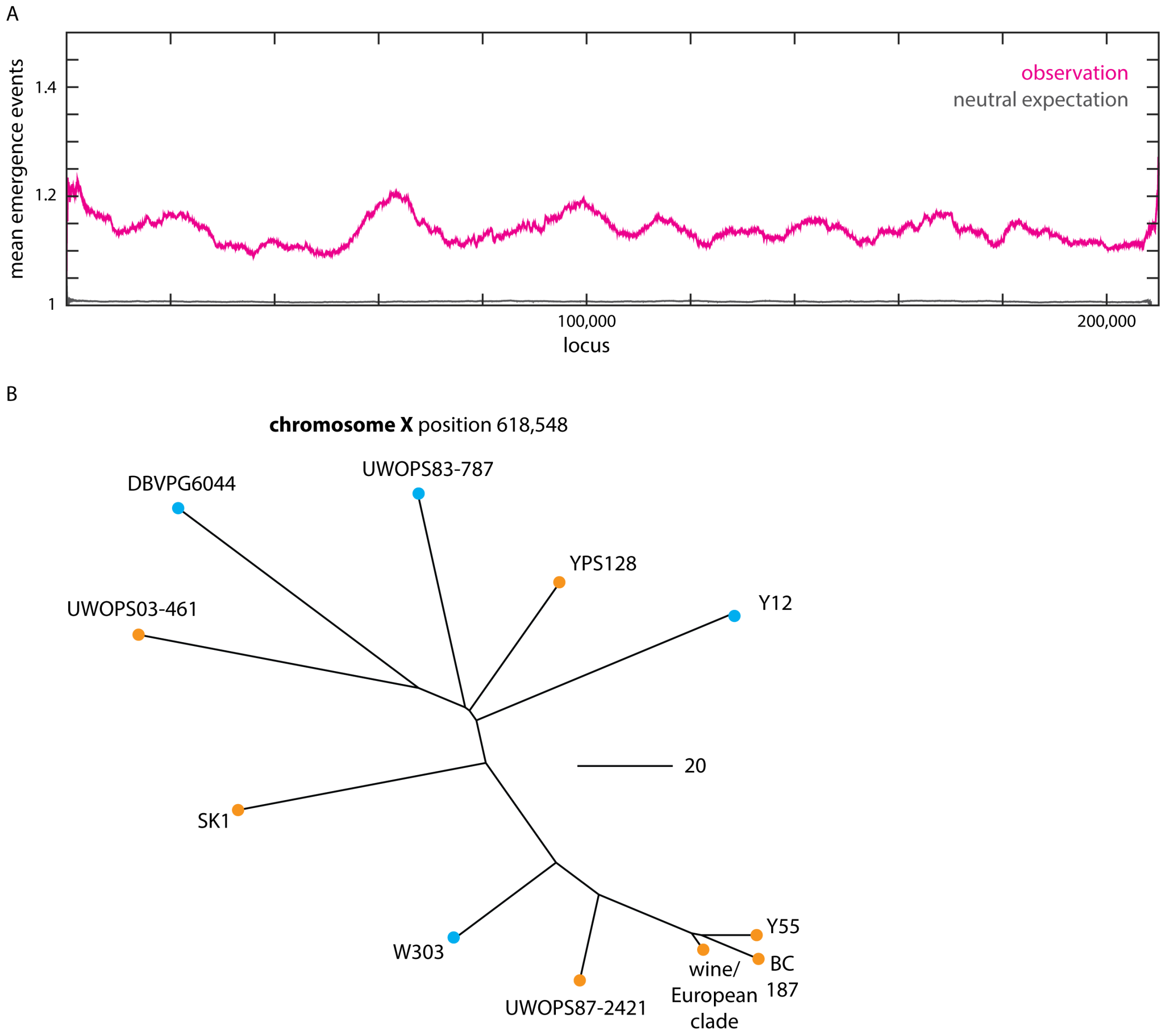

Supplementary Figure 3. (A) Number of inferred multiple emergence events (pink; per 500-variant window) as compared to the neutral expectation (grey) as a function of locus number. **(B)** Example local phylogeny used for emergence inference; for this locus, the algorithm correctly ascertained seven alternate alleles and four apparent independent emergence events on the basis of this tree. Scale bar shows neighbor-joining distance. Source data are provided as a Source Data file.

Supplementary Tables

	**24h**	**48h**	**72h**	**96h**
**Glucose**	10.0 (5)	9.00 (4)	5.00 (5)	6.66 (5)
**Raffinose**	8.57 (4)	8.88 (4)	0.00 (5)	7.00 (4)
**Galactose**	7.50 (5)	1.00 (5)	3.50 (4)	1.11 (5)
**Maltose**	5.45 (4)	2.85 (5)	8.00 (5)	2.85 (5)
**Sucrose**	5.00 (4)	3.33 (5)	6.92 (4)	8.12 (4)
**Glycerol**	4.28 (5)	5.00 (5)	8.57 (5)	4.11 (4)
**Ethanol**	8.33 (5)	4.44 (5)	7.50 (4)	6.25 (5)

Supplementary Table 1. Estimated false discovery rate at the QTL level [mean % FDR (-$\log_{10}$ *p*-value cutoff)] based on regression on N = 10 random permutations of real growth data for each growth condition.

Media component	Concentration
Carbon source (varies)	2% w/v
Yeast nitrogen base	6.7 g/L
Agar (bacteriological)	2% w/v
Complete supplement mixture	
Adenine	10 mg/L
Arginine	50 mg/L
Aspartic acid	80 mg/L
Histidine	20 mg/L
Isoleucine	50 mg/L
Leucine	100 mg/L
Lysine	50 mg/L
Methionine	20 mg/L
Phenylalanine	50 mg/L
Threonine	100 mg/L
Tryptophan	50 mg/L
Tyrosine	50 mg/L
Valine	140 mg/L
Uracil	20 mg/L

Supplementary Table 2. Media composition.

Strain/Plasmid	Genotype	Provenance
RM11**a**	*MATa ho::kanMX ura3Δ0 leu2Δ0*	Lindquist Lab
YJM975**α**	*MATα ho::hygMX uraΔ3::KanMX his3Δ::NatMX*	NCYC

Supplementary Table 3. Parental genotypes.
