# Supplementary material for: Pervasive function and evidence for selection across standing genetic variation in S. cerevisiae
Source: Nat Commun. 2019 Mar 15;10:1222. doi: 10.1038/s41467-019-09166-1 (PMC6420628; doi:10.1038/s41467-019-09166-1)
Supplement: Supplementary file 3 — Description of Additional Supplementary Files [file 41467_2019_9166_MOESM3_ESM.pdf]

Description of Additional Supplementary Files

File Name: Supplementary Data 1
Description: All genetic mapping results.
