# Supplementary figures and images for: Pervasive function and evidence for selection across standing genetic variation in S. cerevisiae
Source: Nat Commun. 2019 Mar 15;10:1222. doi: 10.1038/s41467-019-09166-1 (PMC6420628; doi:10.1038/s41467-019-09166-1)

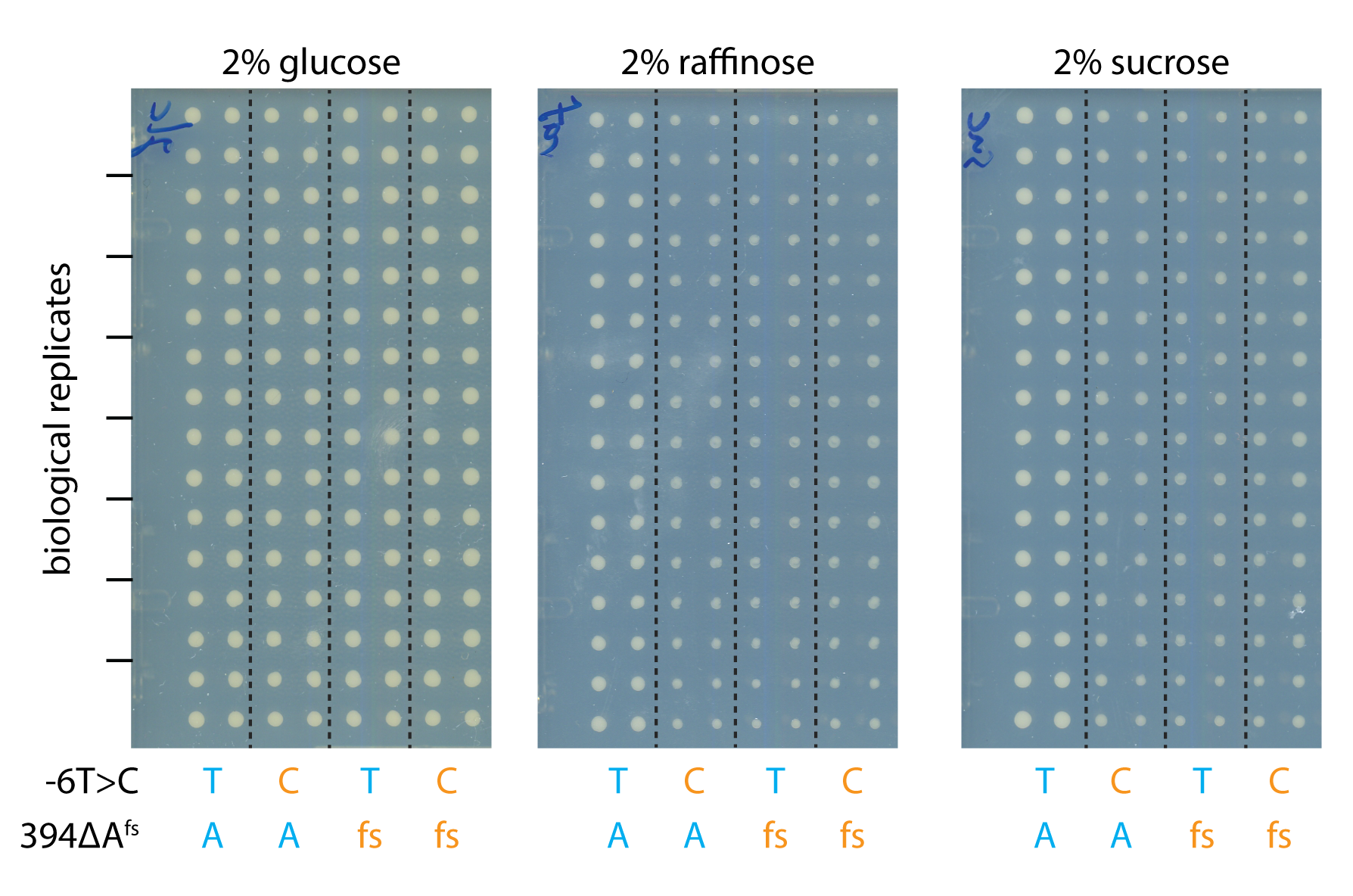

Supplement: Supplementary file 6 — Source Data [file 41467_2019_9166_MOESM6_ESM.zip › rawData/Fig2C.tif]
